# Development of Organelle Replacement Therapy Using a Stearyl-Polyhistidine Peptide against Lysosomal Storage Disease Cells

**DOI:** 10.3390/molecules24162995

**Published:** 2019-08-18

**Authors:** Taiki Hayashi, Riku Okamoto, Tsuyoshi Kawano, Takashi Iwasaki

**Affiliations:** 1 Department of Agricultural Science, Graduate School of Sustainability Science, Tottori University, Tottori 680-8553, Japan; 2 Department of Bioresources Science, Faculty of Agriculture, Tottori University, Tottori 680-8553, Japan

**Keywords:** cell-penetrating peptide, drug delivery system, lysosome, lysosome storage disease, Fabry disease, histidine

## Abstract

We previously reported on a polyhistidine peptide, His16 peptide, as a new cell-penetrating peptide. This peptide is anticipated to be a new carrier for drug delivery systems (DDSs) for targeting intracellular lysosomes because it can transport macromolecules (e.g., liposomes) into these organelles. In the present study, we examined the application of His16 peptide as a DDS carrier against lysosomal storage disease (LSD) cells. LSDs are metabolic disorders caused by loss of specific lysosomal enzymes. For the treatment of LSD cells, we devised a system designated organelle replacement therapy (ORT). ORT is a strategy for transporting exogenous lysosomes containing all kinds of lysosomal enzymes from normal cells into endogenous lysosomes in LSD cells using His16 peptide. To develop the ORT system, we prepared His16 peptide-modified healthy lysosomes (His16-Lyso) by insertion of a stearyl-His16 peptide into a hydrophobic region in the lysosomal membrane. His16-Lyso showed cellular uptake and localization to endogenous lysosomes in LSD cells. His16-Lyso also restored the proliferation of LSD cells, which otherwise showed slower proliferation than normal cells. These results suggested that His16-Lyso replenished deficient lysosomal enzymes in LSD cells. The results further suggest that His16-Lyso are promising candidates as a treatment tool for LSD cells and to establish a foundation for ORT.

## 1. Introduction

Cell-penetrating peptides (CPPs) are widely studied as carriers for drug delivery systems (DDSs) because they can transport various sizes of cargos into living cells [1,2]. Previously, we reported a polyhistidine peptide, His16 peptide (HHHHHHHHHHHHHHHH–NH_2_), as a new CPP. In contrast to conventional CPPs consisting of arginine/lysine-rich sequences, His16 peptide contains only histidine residues. However, His16 peptide showed higher cellular uptake and stability in serum than conventional CPPs [3]. Furthermore, His16 peptide can transport diverse sizes of payloads—such as small compounds, proteins, and liposomes—into mammalian cells. Another feature of His16 peptide is lysosomal accumulation. Our previous study showed that His16 peptide fused to both small molecules and macromolecules localized to lysosomes in mammalian cells [3]. These features led us to consider the application of His16 peptide as a DDS carrier targeting intracellular lysosomes, and we subsequently reported a lysosome-targeting DDS using His16 peptide-modified liposomes [4]. Therefore, His16 peptide is anticipated to be a new carrier for DDSs targeting intracellular lysosomes.

Lysosomes are essential subcellular organelles that digest and recycle many biomolecules to control cellular metabolism [5]. Lysosomes contain about 60 lysosomal enzymes involved in the hydrolysis of macromolecules, including peptides, nucleic acids, carbohydrates, and lipids [6]. These lysosomal enzymes are synthesized in the endoplasmic reticulum based on nuclear genes. The synthesized lysosomal enzymes are tagged explicitly with mannose 6-phosphate (M6P) and are imported to lysosomes via the Golgi apparatus [7]. However, mutations in genes for lysosomal enzymes are responsible for more than 50 different human genetic disorders, known as lysosomal storage diseases (LSDs). LSDs are metabolic disorders caused by the loss of specific lysosomal enzymes. LSDs are associated with various neurodegenerative disorders, cancers, cardiovascular diseases, and aging-related diseases [8,9].

Enzyme replacement therapy (ERT) is a method for supplying LSD cells with deficient enzymes, and is well known as a promising treatment for LSDs [10,11]. Most lysosomal enzymes used in ERT are modified with M6P, a targeting signal for transportation to lysosomes. M6P-tagged exogenous lysosomal enzymes bind to M6P receptors on the cell surface and are transported to endogenous lysosomes via late endosomes [12]. Therefore, high-density M6P modifications are required for conventional ERT. However, few effective expression methods can yield recombinant lysosomal enzymes with multiple M6P modifications. Consequently, conventional ERT has limitations on the variety of available lysosomal enzymes that can be delivered to endogenous lysosomes. As of 2019, despite there being more than 50 lysosomal enzymes related to the same number of LSDs, only 12 recombinant lysosomal enzymes (agalsidase alfa, agalsidase beta, alglucosidase alfa, laronidase, elosulfase alfa, galsulfase, idursulfase, imiglucerase, sebelipase alfa, taliglucerase alfa, velaglucerase alfa, and vestronidase alfa-vjbk) have been approved by the US Food and Drug Administration and are used in ERT against a few types of LSDs (Gaucher disease, Fabry disease (FD), Pompe disease, and mucopolysaccharidosis I, II, IV, VI, and VII) [13,14]. Thus, conventional M6P-dependent ERT has a problem of low availability against most LSDs.

**Figure 1:**
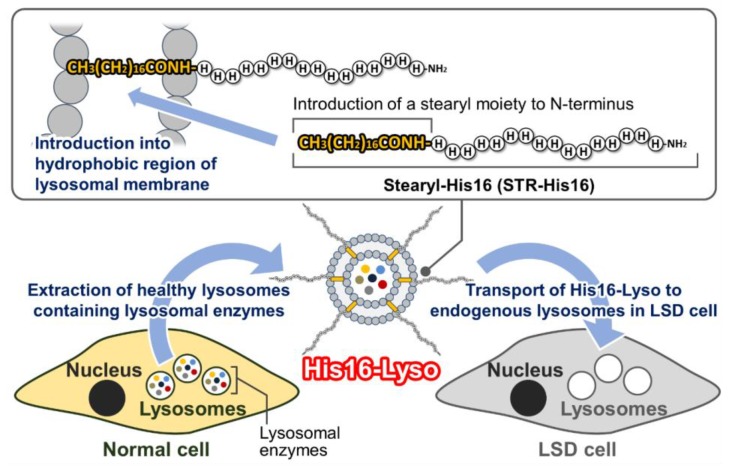
Scheme for organelle replacement therapy (ORT) using His16 peptide-modified lysosomes (His16-Lyso). Healthy lysosomes containing lysosomal enzymes are completely extracted from normal cells. The extracted lysosomes are modified with His16 peptide by insertion of the stearyl-His16 peptide into the hydrophobic region in the lysosomal membrane. His16-Lyso become internalized into LSD cells and are transported to endogenous lysosomes by His16 peptide functions.

To address this problem that is associated with conventional ERT, we examined the development of M6P-independent ERT using His16 peptide. In the present study, we devised the new concept of organelle replacement therapy (ORT) as a new ERT. ORT is a strategy for transporting intact healthy lysosomes derived from normal cells (non-LSD cells) to endogenous lysosomes in LSD cells using His16 peptide (Figure 1).

Since whole lysosomes contain all kinds of active lysosomal enzymes, ORT is expected to avoid some limitations associated with conventional ERT. To develop ORT in the present study, we extracted healthy lysosomes from human fibrosarcoma HT1080 cells. The HT1080 cell line is used for the expression of recombinant lysosomal enzymes for ERT [15,16,17]. Therefore, lysosomes derived from HT1080 cells are expected to be highly biocompatible for ERT. The extracted lysosomes were modified with His16 peptide by insertion of the stearyl-His16 peptide (STR-His16) into the hydrophobic region in the lysosomal membrane. Stearylation was well used for insertion of CPPs on the surface of lipid bilayer membrane retaining cellular uptake ability. Stearyl-CPPs were inserted to the surface of the lipid bilayer membrane of liposomes, and CPPs-modified liposomes showed cellular uptake in various studies [18,19]. Lysosomes also have lipid bilayer membranes similarly to liposomes. Therefore, we used stearylation of the His16 peptide as a means to allow its insert into the surface of lysosomes and facilitate their transport into cells. The resulting His16 peptide-modified lysosomes were designated His16-Lyso. Here, we first established a method for ORT using His16-Lyso in a model experiment using human fibrosarcoma HT1080 cells as non-LSD cells. Subsequently, we confirmed the ERT efficacy of ORT in a demonstration experiment using FD patient fibroblasts as a type of LSD cell.

## 2. Results and Discussion

### 2.1. Preparation of Lysosomes Showing Red Fluorescence

**Figure 2:**
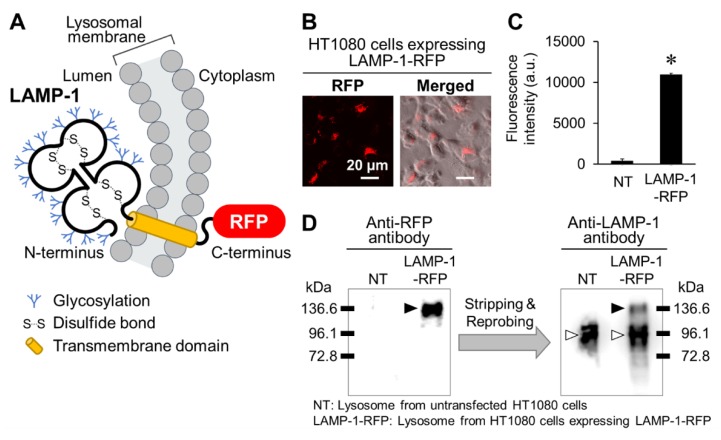
Construction of lysosomes expressing LAMP-1-RFP in human fibrosarcoma HT1080 cells. (**A**) Schematic representation of LAMP-1-RFP in the lysosomal membrane. (**B**) HT1080 cells expressing LAMP-1-RFP were established by transfection of a LAMP-1-RFP expression plasmid. Red fluorescence indicates LAMP-1-RFP. (**C**) Lysosomes were extracted from untransfected and transfected HT1080 cells. The extracted lysosomes were captured, and their red fluorescence intensity was measured with a multiplate reader. (**D**) The extracted lysosomes were analyzed by Western blotting using anti-RFP and anti-LAMP-1 antibodies. Each PVDF membrane was initially probed with the anti-RFP antibody and then stripped and reprobed with the anti-LAMP-1 antibody. The black and white arrowheads indicate LAMP-1-RFP and native LAMP-1, respectively.

To examine whether His16-Lyso showed cellular uptake into cells, lysosomes expressing red fluorescent protein (RFP) were prepared. For this, we constructed human fibrosarcoma HT1080 cells stably expressing lysosome-associated membrane glycoprotein-1 (LAMP-1) fused to RFP by transfection with a pCMV-LAMP-1-RFP expression plasmid (Figure 2A). LAMP-1 is a major protein component of the lysosomal membrane and a lysosomal marker protein [20]. In our result, red fluorescence derived from LAMP-1-RFP was recognized in the transfected cells (Figure 2B). Subsequently, we isolated lysosomes from untransfected and transfected HT1080 cells. The lysosomes isolated from transfected cells showed red fluorescence (Figure 2C). Thereafter, the LAMP-1 and RFP present in the lysosome samples were detected by Western blotting analysis. RFP was only detected at approximately 136.6 kDa in lysosomes from HT1080 cells expressing LAMP-1-RFP. LAMP-1 was detected at approximately 96.1 kDa in lysosomes from both untransfected and transfected HT1080 cells, while a band for LAMP-1 at approximately 136.6 kDa was specifically detected in lysosomes from transfected HT1080 cells (Figure 2D). LAMP-1 is composed of 382 amino acids, corresponding to about 42 kDa [20]. However, the apparent size of LAMP-1 was converted to approximately 100 kDa by processing for high glycosylation (Figure 2A) [21]. These results demonstrate that the proteins at approximately 96.1 kDa and 136.6 kDa are intact LAMP-1 and LAMP-1-RFP, respectively. Thus, we successfully established lysosomes showing red fluorescence.

### 2.2. Cellular Uptake and Intracellular Distribution of His16-Lyso in HT1080 Cells

**Figure 3:**
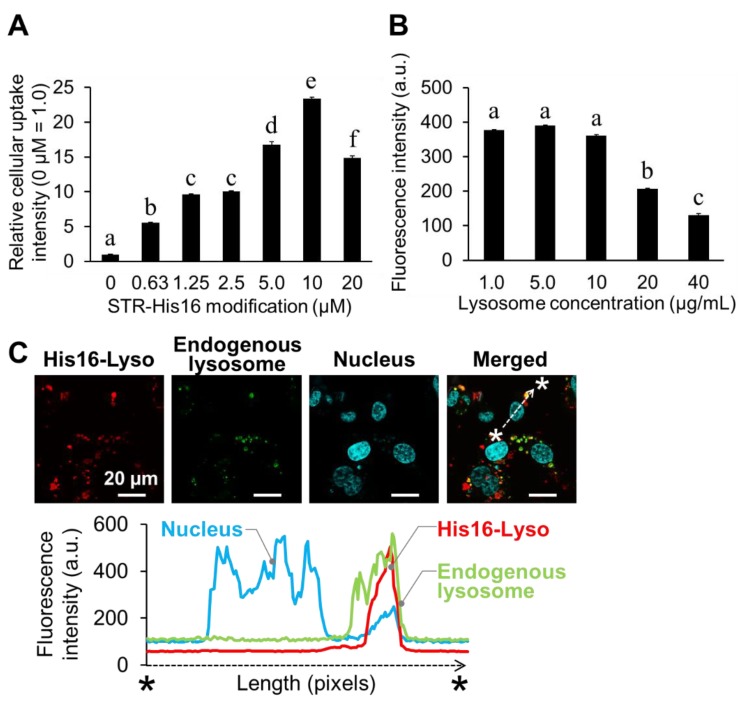
Model experiment for ORT using human fibrosarcoma HT1080 cells. (**A**) The optimal stearyl (STR)-His16 modification ratio for cellular uptake of His16-Lyso was determined. His16-Lyso were prepared by incorporating 0–20 µM STR-His16 peptide into 1.0 µg/mL lysosomes expressing LAMP-1-RFP. The relative cellular uptake of His16-Lyso were evaluated by the mean fluorescence intensity in a flow cytometric analysis. (**B**) The optimal lysosome concentration for cellular uptake of His16-Lyso was determined. His16-Lyso were prepared by incorporating 10 µM STR-His16 peptide into 1.0–40 µg/mL lysosomes expressing LAMP-1-RFP. Data represent means ± SD. Different letters indicate significant differences (*p* < 0.05, Tukey’s test). (**C**) Intracellular distribution of His16-Lyso in HT1080 cells. Red, green, and cyan fluorescence indicates His16-Lyso expressing LAMP-1-RFP, endogenous lysosomes stained with LysoTracker Green, and nuclei stained with Hoechst 33258, respectively. The distributions of His16-Lyso, endogenous lysosomes, and nuclei were quantified by measurement of the fluorescence intensities along the line between the two asterisks.

In our previous study, His16 peptide showed its highest cellular uptake in HT1080 cells [3]. Therefore, the HT1080 cell line was initially used to examine the cellular uptake of His16-Lyso in a model experiment. His16-Lyso were prepared by insertion of various concentrations of STR-His16 peptide in the hydrophobic region in the lysosomal membrane. The cellular uptake of His16-Lyso was analyzed by flow cytometric analysis and the optimal modification concentration of STR-His16 was determined. As expected, the cellular uptake of His16-Lyso increased in an STR-His16 dose-dependent manner (Figure 3A). His16-Lyso prepared with 10 µM STR-His16 showed the maximum cellular uptake. Therefore, we determined that 10 µM STR-His16 was the optimal modification concentration. The optimal lysosome concentration for cellular uptake of His16-Lyso was also determined. Constant cellular uptake of His16-Lyso was observed for lysosome concentrations of 1.0–10 µg/mL, while His16-Lyso prepared with 5 µg/mL lysosomes showed slightly greater cellular uptake (Figure 3B). However, cellular uptake of His16-Lyso decreased with lysosome concentrations above 20 µg/mL. These results can be explained by a decrease in STR-His16 ratio per lysosome. Therefore, we determined that 5 µg/mL lysosomes were the optimal concentration for cellular uptake of His16-Lyso prepared with 10 µM STR-His16, and these conditions were used in subsequent experiments.

We previously showed that His16 peptide alone and His16 peptide-modified liposomes were localized to intracellular lysosomes in HT1080 cells [3,4]. Therefore, we examined the colocalization between His16-Lyso and endogenous lysosomes. LysoTracker Green and Hoechst 33342 were used as fluorescent staining dyes for endogenous lysosomes and nuclei, respectively. Quantitative fluorescence analysis on confocal laser scanning microscopy (CLSM) images revealed that the red fluorescence derived from His16-Lyso in cells was observed in the same area as green fluorescence from endogenous lysosomes (Figure 3C). These results indicate that His16-Lyso became localized to endogenous lysosomes after cellular uptake in HT1080 cells, similarly to the case of His16 peptide and His16 peptide-modified liposomes.

Furthermore, His16-Lyso containing FITC-dextran also showed cellular uptake in an STR-His16 dose-dependent manner (Figure S2) and colocalization with endogenous lysosomes in HT1080 cells (Figure S3). These results indicate that His16-Lyso transported not only lysosomal membrane protein (LAMP-1-RFP) but also soluble component (FITC-dextran) into endogenous lysosomes. Therefore, His16-Lyso are expected to be function as endogenous lysosome-targeting carriers for lysosomal enzymes. 

### 2.3. Cellular Uptake Pathway for His16-Lyso in HT1080 Cells

**Figure 4:**
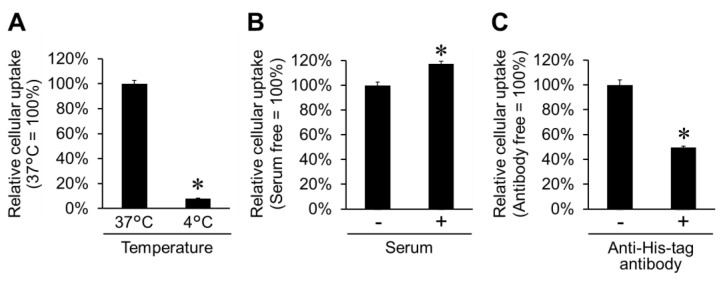
Cellular uptake route of His16-Lyso. Cellular uptake of His16-Lyso (10 µM STR-His16, 5.0 µg/mL lysosomes) in HT1080 cells was determined at 37 °C or 4 °C (**A**), in the presence or absence of 10% serum at 37 °C (**B**), and in the presence or absence of anti-His-tag antibody (1000-fold dilution) at 37 °C (**C**). Cellular uptake was evaluated by the mean fluorescence intensity in a flow cytometric analysis (pH 7.4). Data represent means ± SD. Asterisks indicate significant differences (*p* < 0.05, Student’s *t*-test).

To clarify the cellular uptake pathway for His16-Lyso, the influences of temperature, serum, and anti-His-tag antibody on the cellular uptake of His16-Lyso were examined. Cellular uptake of H16-Lyso was significantly inhibited under a low-temperature condition (4 °C) (Figure 4A). Currently, various CPPs have been reported to penetrate the cell membrane via a temperature-dependent pathway, with inhibition of their cellular uptake under low-temperature conditions [22,23,24]. Our previous study showed that cellular uptake of His16 peptide was inhibited under low-temperature conditions, and the peptide penetrated mammalian cell membranes via a temperature-dependent pathway [3]. Therefore, the cellular uptake of His16-Lyso is also considered to occur via the temperature-dependent pathway.

Interestingly, His16-Lyso showed slightly higher cellular uptake at 37 °C in the presence of serum compared with when serum was absent (Figure 4B). These results suggest that cellular uptake of His16-Lyso was stabilized by the presence of serum. Previously, we reported that cellular uptake of His16 peptide was not affected by serum [3], although serum is known to exhibit ionic interactions with cationic CPPs and dramatically suppress their cellular uptake [25,26]. In another study, we showed that cellular uptake of His16 peptide-modified liposomes was slightly enhanced in the presence of serum [4]. Thus, increased membrane vesicle transport by His16 peptide with serum can explain the observed stable cellular uptake of His16-Lyso in the presence of serum, and this is a useful feature for in vivo ERT.

An anti-His-tag antibody was used to inhibit His16 peptide and establish whether cellular uptake of His16-Lyso was mediated by the peptide. The cellular uptake of His16-Lyso was inhibited by half in the presence of the anti-His-tag antibody at 37 °C (Figure 4C). These results suggest that the antibody masked His16 peptide on the surface of His16-Lyso and inhibited the interaction of His16-Lyso with the cell membrane. However, the cellular uptake of His16-Lyso was not completely inhibited by the anti-His-tag antibody. We speculate that this finding arose because His16 peptide was not completely masked by the anti-His-tag antibody. Steric hindrance among macromolecule antibodies can interfere with the number of anti-His-tag antibodies that bind to the short His16 peptide, and partially exposed histidine residues on His16 peptide may contribute to cellular uptake of His16-Lyso. Our previous study showed that a shorter polyhistidine peptide, His10 peptide, also exhibited some cellular uptake [3]. Also, some serum proteins can show nonspecific binding to antibodies and may reduce the binding of an anti-His-tag antibody to His16 peptide. Nevertheless, these results demonstrate that His16-Lyso were internalized into cells via the His16 peptide-mediated cellular uptake pathway.

The model experiment using the HT1080 cell line demonstrated that His16-Lyso were internalized into cells via the His16 peptide-mediated pathway and became localized to endogenous lysosomes. These results suggest that His16-Lyso have the potential for ERT against LSD cells. Therefore, we subsequently investigated the cellular uptake and intracellular localization of His16-Lyso in a demonstration experiment using FD patient fibroblasts.

### 2.4. Cellular Uptake and Intracellular Distribution of His16-Lyso in FD Patient Fibroblasts

**Figure 5:**
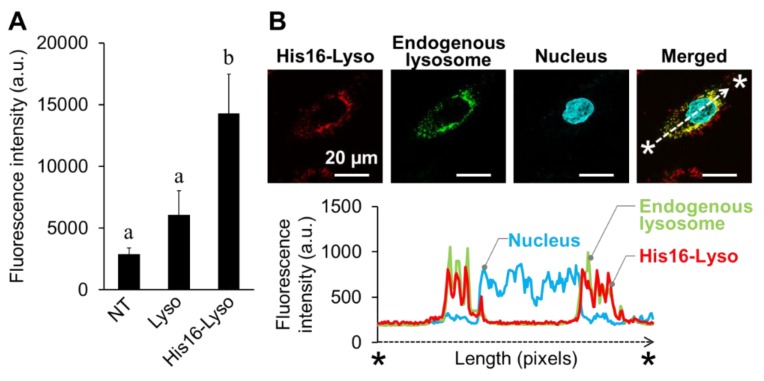
Demonstration experiment for ORT using Fabry disease (FD) patient fibroblasts. (**A**) Cellular uptake of His16-Lyso (10 µM STR-His16, 5.0 µg/mL lysosomes) was evaluated by the mean fluorescence intensity in fluorescence microplate reader analysis (pH 7.4). NT and Lyso mean nontreated condition and unmodified lysosomes, respectively. Data represent means ± SD. Different letters indicate significant differences (*p* < 0.05, Tukey’s test). (**B**) Intracellular distribution of His16-Lyso in FD patient fibroblasts. Red, green, and cyan fluorescence indicates His16-Lyso, endogenous lysosomes stained with LysoTracker Green, and nuclei stained with Hoechst 33258, respectively. The distributions of His16-Lyso, endogenous lysosomes, and nuclei were quantified by measuring the fluorescence intensities along the line between the two asterisks.

The cellular uptake of His16-Lyso in FD patient fibroblasts was analyzed by fluorescence intensity measurements using a multiplate reader because the nonhomogeneous sizes of cells made it difficult to carry out flow cytometric analyses. Compared with nontreated and unmodified lysosome-treated FD patient fibroblasts, His16-Lyso-treated cells showed higher red fluorescence intensity (Figure 5A).

The intracellular distribution of His16-Lyso in FD patient fibroblasts was analyzed by CLSM. Specifically, we examined the colocalization of His16-Lyso and endogenous lysosomes as described above. We observed that red fluorescence derived from His16-Lyso was present in the cells and colocalized with green fluorescence from endogenous lysosomes. A quantification analysis confirmed the colocalization of the fluorescence signals from His16-Lyso and endogenous lysosomes (Figure 5B).

These results indicate that His16-Lyso were internalized by FD patient fibroblasts and became localized to endogenous lysosomes. Thus, we concluded that His16-Lyso are available for supplementation of lysosomal enzymes to endogenous lysosomes in FD patient fibroblasts.

### 2.5. ERT Efficacy of His16-Lyso in FD Patient Fibroblasts

**Figure 6:**
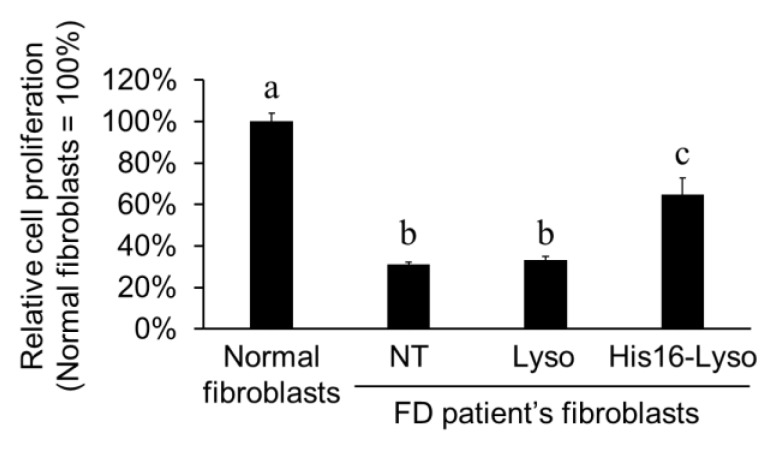
Enzyme replacement therapy (ERT) efficacy of His16-Lyso against FD patient fibroblasts. The FD patient fibroblasts were treated with or without His16-Lyso (10 µM STR-His16, 5.0 µg/mL lysosomes) for 72 h. NT and Lyso mean nontreated condition and unmodified lysosomes from HT1080 cells, respectively. Cell proliferation was determined by the water-soluble tetrazolium salt (WST) assay. Data represent means ± SD. Different letters indicate significant differences (*p* < 0.05, Tukey’s test).

FD is caused by loss of galactosidase alfa (GLA), a type of lysosomal enzyme [27]. GLA-knockdown cells were reported to show slower cell proliferation than normal cells [4,27]. Therefore, we evaluated the ERT efficacy of His16-Lyso against FD patient fibroblasts by measuring cell proliferation recovery. Compared with normal fibroblasts, FD patient fibroblasts showed decreased cell proliferation. Unmodified lysosome treatment showed no recovery of cell proliferation. However, His16-Lyso treatment increased the cell proliferation of FD patient fibroblasts by about 2.1-fold compared with nontreated cells (Figure 6). Furthermore, treatment using a high concentration of His16-Lyso (25 µg/mL lysosomes) completely restored cell proliferation in FD patient fibroblasts (Figure S4). On the other hand, His16 or STR-His16 peptides alone show no effect on the cell proliferation of FD patient fibroblasts (Figure S5). These results suggest that His16-Lyso replenished GLA in FD patient fibroblasts and consequently restored the cell proliferation. These results further indicate that His16-Lyso showed ERT efficacy against FD patient fibroblasts. In the demonstration experiment, we confirmed successful ORT using His16-Lyso.

We have previously succeeded in restoring the cell proliferation of GLA-knockdown cells using His16 peptide-modified liposomes containing GLA [4]. However, the advantage of this study is a simple process to prepare vesicles containing lysosomal enzymes. In previous our work, it was necessary to prepare liposomes containing lysosomal enzymes to deliver lysosomal enzymes into endogenous lysosomes. However, specific technical skills and devices are required for the preparation of liposome-containing enzymes. On the other hand, we used isolated lysosomes here as vesicles containing lysosomal enzymes, and prepared them by simple processes (i.e., cell lysis and centrifugation). This unique concept places this study in a superior position to our previous work.

## 3. Materials and Methods

### 3.1. Peptide Synthesis and Purification

The STR-His16 peptide was purchased from Biologica Co. (Shanghai, China). His16 peptide was synthesized by solid-phase peptide synthesis, and STR-His16 peptide was prepared by treatment of the His16 peptide on resins with stearic acid (C_17_H_35_COOH) and diisopropylcarbodiimide in the presence of N-hydroxybenzotriazole. STR-His16 peptide with a stearyl moiety at the N-terminus and an amide group at the C-terminus was purified by high-performance liquid chromatography (HPLC) using a Waters 2489 UV/visible detector and 1524 binary pump (Waters, Milford, MA, USA) and a reversed phase COSMOSIL 5C_18_-MS-II column (10 mm × 250 mm; Nacalai Tesque, Kyoto, Japan) as previously described [4]. The column was run for 40 min at 3 mL/min with a linear gradient from 0% to 80% (*v*/*v*) acetonitrile in water containing 0.1% (*v*/*v*) trifluoroacetic acid. The purity of STR-His16 peptide was calculated from peak areas of HPLC chart, and final purity of the peptide was >95% (Figure S1A). The molecular masses were confirmed by matrix-assisted laser desorption/ionization time-of-flight mass spectrometry (AutoFlex II; Bruker Daltonics, Billerica, MA, USA) (Figure S1B).

### 3.2. Cell Culture

Human fibrosarcoma HT1080 cells were purchased from Riken BRC Cell Bank (Ibaraki, Japan) and cultured in Eagle’s minimal essential medium (Wako, Osaka, Japan). Human normal and FD patient skin fibroblasts, immortalized by transfection of an SV40 large T cDNA expression vector (pET321-T), were kindly supplied by Dr. Katsumi Higaki (Tottori University) and cultured in Dulbecco’s modified Eagle’s medium (Wako). All media contained 10% fetal bovine serum (FBS) (*v/v*), 100 µg/mL streptomycin, 100 U/mL penicillin, and 250 ng/mL amphotericin B. Cells were maintained at 37 °C in a humidified 5.0% CO_2_ incubator and subcultured every 3 days.

### 3.3. Preparation of HT1080 Cells Stably Expressing LAMP-1-RFP

We prepared HT1080 cells stably expressing RFP-fused LAMP-1, in which RFP was fused to the C-terminus of LAMP-1 (Figure 2A). HT1080 cells were plated in 24-well plates at 10 × 10^4^ cells/well in a final volume of 250 µL and incubated for 24 h at 37 °C under 5.0% CO_2_. After complete adhesion, the cells were transfected with a LAMP-1-RFP expression plasmid (pCMV-LAMP-1-RFP) using Xfect Transfection Reagent (TaKaRa Bio, Shiga, Japan) for 4 h. The culture medium was replaced with fresh medium, and the cells were incubated for 48 h at 37 °C under 5.0% CO_2_. Thereafter, the cells were treated with G418 (400 µg/mL) for antibiotic selection of HT1080 cells stably expressing LAMP-1-RFP. 

### 3.4. Isolation of Lysosomes

Isolation of lysosomes was performed as previously described [28]. HT1080 cells were cultured to confluency in T-75 flasks, washed with phosphate-buffered saline (PBS), and harvested with 0.05% trypsin/0.53 mM EDTA. Thereafter, the cells were centrifuged at 500*g* for 5 min and washed twice with PBS. After removal of the supernatant, the cells were resuspended in 1.5 mL of extraction buffer (10 mM Tris-HCl pH 7.4 containing 0.25 M sucrose) and homogenized in a Potter-type homogenizer (Digital Homogenizer; Iuchi, Osaka, Japan) with 50 strokes at 1440 rpm. The cell lysate was centrifuged (1000*g*) for 10 min at 4 °C to remove cell debris. The supernatant was centrifuged (15,000*g*) for 15 min at 4 °C, and the pellet was collected in 1 mL of PBS as the lysosome fraction. The concentration of isolated lysosomes was determined using a Protein Assay BCA Kit (Wako).

### 3.5. Preparation of His16-Lyso

His16-Lyso were prepared by insertion of STR-His16 into the hydrophobic region in the lysosomal membrane. Isolated lysosomes were incubated with various concentrations of STR-His16 for 1 h at 37 °C in the dark.

### 3.6. Cellular Uptake of His16-Lyso

#### 3.6.1. Flow Cytometric Analysis

Cellular uptake of His16-Lyso was determined by flow cytometric analysis as previously described [3]. HT1080 cells were seeded onto 12-well plates at a density of 10 × 10^4^ cells/well in a final volume of 500 µL and incubated for 24 h at 37 °C under 5.0% CO_2_. The culture medium was replaced with fresh medium (500 µL/well) containing His16-Lyso expressing LAMP-1-RFP, and the cells were incubated for 24 h. Thereafter, the cells were washed twice with PBS, harvested with 0.05% trypsin/0.53 mM EDTA, and suspended in 500 µL of FACS buffer (PBS pH 7.4 containing 2% FBS). Intracellular uptake of His16-Lyso was evaluated by flow cytometric analysis with a BD FACSCanto II (Becton, Dickinson and Company, New Jersey, NJ, USA) using 488 nm laser excitation and a 575/25 bandpass filter. Approximately 1.0 × 10^4^ cells were collected per specimen with three repetitions.

#### 3.6.2. Fluorescence Microplate Reader Analysis

Cellular uptake of His16-Lyso was determined by fluorescence microplate reader analysis. Normal and FD patient fibroblasts were seeded onto 96-well plates at a density of 1.0 × 10^4^ cells/well in a final volume of 100 µL and incubated for 24 h at 37 °C under 5.0% CO_2_. The culture medium was replaced with fresh medium (100 µL/well) containing His16-Lyso expressing LAMP-1-RFP, and the cells were incubated for 24 h. The cells were washed twice with PBS and disrupted in RIPA buffer (40 mM Tris-HCl pH 7.4, 300 mM NaCl, 0.2% SDS, 2% Nonidet P-40, 1% deoxycholic acid, 4 mM EDTA). The red fluorescence intensity in lysates was measured at an excitation wavelength 550 nm and an emission wavelength 590 nm using an Infinite 200 Pro multiplate reader (Tecan, Männedorf, Switzerland).

### 3.7. CLSM Analysis

CLSM analysis was performed as previously described [3]. Cells were seeded onto multiwell glass-bottom dishes (Matsunami Industries, Osaka, Japan) at a density of 2.0 × 10^4^ cells/well in a final volume of 100 µL and incubated for 24 h at 37 °C under 5% CO_2_. The expression of LAMP-1-RFP was observed with excitation wavelength of 555 nm and emission wavelength of 580 nm. The intracellular distribution of His16-Lyso was also determined by CLSM. LysoTracker Green (Life Technologies, Carlsbad, CA, USA) and Hoechst 33342 (Dojindo, Kumamoto, Japan) were used as an organelle marker for lysosomes and a nuclear stain, respectively. After complete adhesion, His16-Lyso were added and the cells were incubated for 24 h. The culture medium was replaced with fresh medium, and Hoechst 33342 was added to the cells and incubated for 1 h. After two washes with PBS, fresh medium containing 500 nM LysoTracker Green was added, and the cells were incubated for 2 h. The intracellular distribution of His16-Lyso was observed and fluorescence images were acquired using a FluoView CLSM (Olympus, Tokyo, Japan).

### 3.8. Cell Proliferation Assay (WST Assay)

To examine the therapeutic efficacy of His16-Lyso on LSD cells, FD patient fibroblasts were seeded onto 96-well plates at 5.0 × 10^3^ cells/well in a final volume of 100 µL and incubated for 24 h at 37 °C under 5.0% CO_2_. After pre-incubation in culture medium containing His16-Lyso for 72 h at 37 °C under 5.0% CO_2_, the culture medium was replaced with 100 µL of fresh medium containing 10% Cell Counting Kit-8 reaction solution (Dojindo, Kumamoto, Japan) and the cells were incubated for 4 h at 37 °C under 5.0% CO_2_. Cell proliferation was determined by measuring the absorbance at 450 nm using the Infinite 200 Pro multiplate reader.

### 3.9. Statistical Analysis

Differences between two groups were analyzed by Student’s *t*-test with a two-tailed distribution, while differences among multiple groups were evaluated by Tukey’s test. All statistical analyses were carried out with add-in software for Excel (Excel Statistics 2010; SSRI, Tokyo, Japan). A value of *p* < 0.05 was considered statistically significant.

## 4. Conclusions

In conclusion, we have established a method for ORT using His16-Lyso. In the model experiment, His16-Lyso showed cellular uptake and intracellular localization to endogenous lysosomes in human fibrosarcoma HT1080 cells. We also confirmed successful ORT against LSD cells. In the demonstration experiment, His16-Lyso were internalized into FD patient fibroblasts and restored cell proliferation. These findings suggest that His16-Lyso showed ERT efficacy by replenishing lysosomal enzymes in endogenous lysosomes in LSD cells. The findings further suggest that ORT is a new strategy, and His16-Lyso are promising candidates for the treatment of LSDs. ORT using His16-Lyso is expected to be useful for LSDs other than FD, and we intend to support this expectation through future studies.

## Supplementary Materials

The following are available online. Figure S1: HPLC profile and the mass spectrum of STR-His16 peptide, Figure S2: Cellular uptake of His16-Lyso containing FITC-dextran in HT1080 cells, Figure S3: Intracellular distribution of His16-Lyso containing FITC-dextran in HT1080 cells, Figure S4: ERT efficacy of a large amount of His16-Lyso against FD patient fibroblasts, Figure S5: Cell proliferation of FD patient fibroblasts treated with His16 or STR-His16 peptides.
