# Supplementary material for: Development of Organelle Replacement Therapy Using a Stearyl-Polyhistidine Peptide against Lysosomal Storage Disease Cells
Source: Molecules. 2019 Aug 18;24(16):2995. doi: 10.3390/molecules24162995 (PMC6720886; doi:10.3390/molecules24162995)
Supplement: Supplementary file 1 [file molecules-24-02995-s001.pdf]

molecules

MDPI

*Supplementary materials for*

# Development of Organelle Replacement Therapy using a Stearyl-Polyhistidine Peptide against Lysosomal Storage Disease Cells

**Taiki Hayashi [1], Riku Okamoto [2], Tsuyoshi Kawano [1] and Takashi Iwasaki [1,*]**

[1] Department of Agricultural Science, Graduate School of Sustainability Science, Tottori University, Tottori 680-8553, Japan; t.hayashi56@gmail.com (T.H.); kawano@tottori-u.ac.jp (T.K.); itaka@tottori-u.ac.jp (T.I.)

[2] Department of Bioresources Science, Faculty of Agriculture, Tottori University, Tottori 680-8553, Japan; riku0529@gmail.com (R.O.)

* Correspondence: itaka@tottori-u.ac.jp; Tel.: +81-857-31-5458

## 1. HPLC profile and the mass spectrum of STR-His16 peptide

The purity of STR-His16 peptide was checked by HPLC before use for the experiment. HPLC profile showed that the final purity of STR-His16 peptide was >95%; it calculated from peak areas (Figure S1A). The molecular masses of the final purified peptide, eluted at 27.22 min in HPLC profile, were measured by matrix-assisted laser desorption/ionization time-of-flight mass spectrometry (MALDI-TOF-MS). The molecular masses of STR-His16 peptide (2477.72 Da) were confirmed as molecular ions $[M+Na]^+$ (*m/z* 2500.74) and $[M+K]^+$ (*m/z* 2516.79) (Figure S1B).

**A**

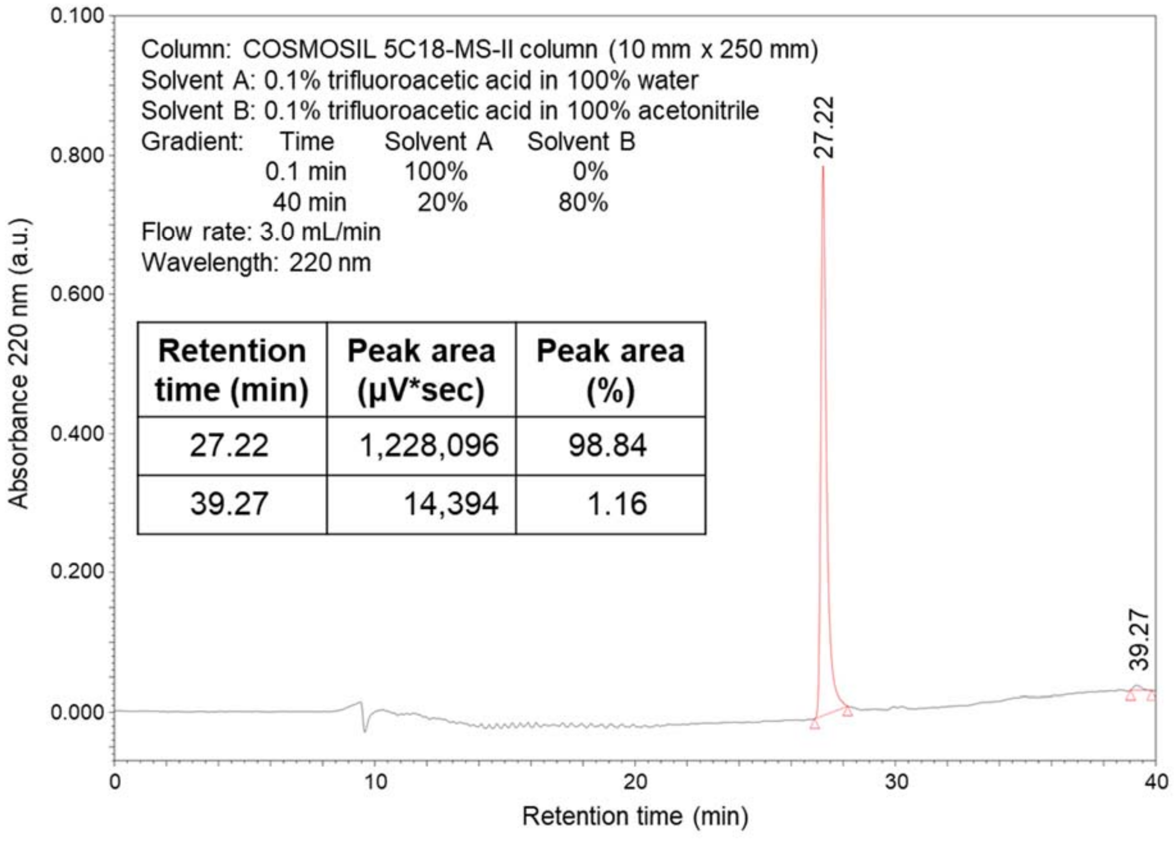

B

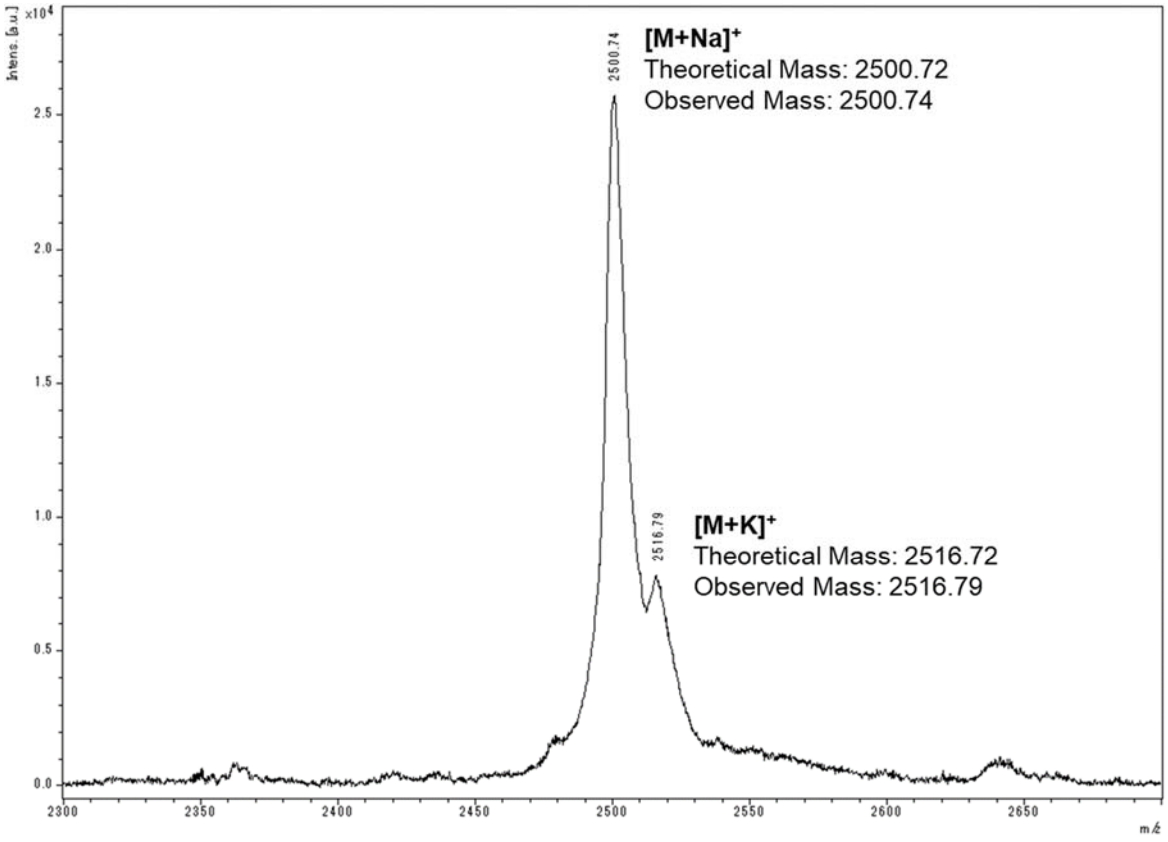


**Figure S1.** HPLC profile and the mass spectrum of STR-His16 peptide. **(A)** HPLC was performed using a Waters 2489 UV/visible detector and 1524 binary pump and a reversed-phase COSMOSIL 5C18-MS-II column (10 mm × 250 mm; Nacalai Tesque). The column was run for 40 min at 3 mL/min with a linear gradient from 0% to 80% (v/v) acetonitrile in water containing 0.1% (v/v) trifluoroacetic acid. **(B)** The mass spectrum of STR-His16 peptide was measured by MALDI-TOF-MS using AutoFlex II (Bruker Daltonics) in positive detection mode using $\alpha$-cyano-4-hydroxycinnamic acid as the matrix.

## 2. Cellular uptake of His16-Lyso containing FITC-dextran in HT1080 cells

HT1080 cells were treated with FITC-dextran (3 mg/mL) for 1 h and FITC-dextran was loaded into lysosomes. Then, lysosomes containing FITC-dextran were isolated from HT1080 cells and modified with STR-His16 peptide. The His16-Lyso containing FITC-dextran was prepared by the insertion of various concentrations of STR-His16 peptide and cellular uptake in HT1080 cells was analyzed by flow cytometric analysis. As a result, the cellular uptake of His16-Lyso containing FITC-dextran increased in an STR-His16 dose-dependent manner (Figure S2), as well as that of His16-Lyso expressing LAMP-1-RFP (Figure 3A). This result indicates that His16-Lyso transported not only lysosomal membrane protein (LAMP-1-RFP) but also soluble component (FITC-dextran) into cells. Figure S2 also suggests that isolated exogenous lysosomes remained intact membrane structure during incubation with HT1080 cells. If lysosomal membrane structure was disrupted during incubation, FITC-dextran was leaked from exogenous lysosomes and transported into cells via endocytosis. However, unmodified lysosomes containing FITC-dextran showed little cellular uptake, and this result suggests exogenous lysosomes were intact during incubation.

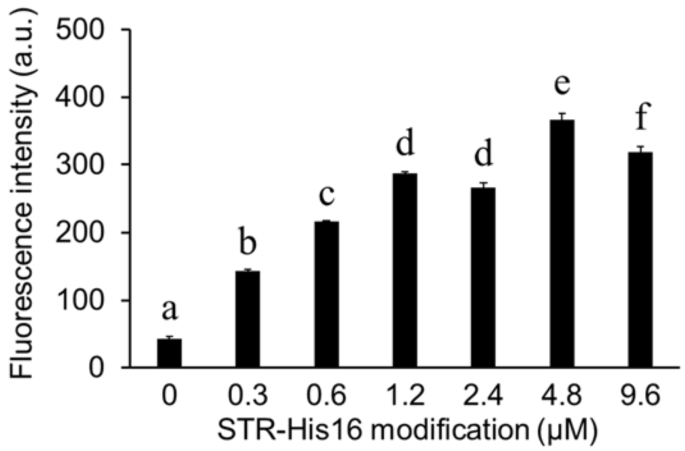


**Figure S2.** Cellular uptake of His16-Lyso containing FITC-dextran in HT1080 cells. His16-Lyso was prepared by incorporating 0–9.6 μM STR-His16 peptide into 1 μg/mL lysosomes containing FITC-dextran. HT1080 cells were incubated with His16-Lyso containing FITC-dextran in culture medium for 24 h at 37°C under 5.0% $CO_2$, and cellular uptake was evaluated by the mean fluorescence intensity in a flow cytometric analysis. Data represent means ± SD. Different letters indicate significant differences ($p < 0.05$, Tukey's test).

### 3. Intracellular distribution of His16-Lyso containing FITC-dextran in HT1080 cells

HT1080 cells were treated with rhodamine-dextran (3 mg/mL) for 1 h, and rhodamine-dextran was preloaded into endogenous lysosomes. After washing with PBS, the HT1080 cells were treated with His16-Lyso containing FITC-dextran and incubated for 24 h. Then, colocalization between His16-Lyso containing FITC-dextran and endogenous lysosomes stained with rhodamine-dextran was assessed. Quantitative fluorescence analysis on CLSM images revealed that green fluorescence derived from His16-Lyso in cells was observed in the same area as red fluorescence from endogenous lysosomes (Figure S3). This result indicates that His16-Lyso containing FITC-dextran became localized to endogenous lysosomes after cellular uptake in HT1080 cells, as well as His16-Lyso expressing LAMP-1-RFP (Figure 3C). This result also indicates that soluble component (FITC-dextran) contained in exogenous His16-Lyso was transported into endogenous lysosomes.

We speculated that His16-Lyso was disrupted and component was released to endosomes during transport to endogenous lysosomes after cellular uptake. Therefore, disrupted His16-Lyso were also speculated not to be detected by LysoTracker. Figure 3C showed partial compartments of His16-Lyso expressing LAMP-1-RFP were not stained with LysoTracker green. However, it is difficult to understand the intracellular behavior of soluble component in His16-Lyso from Figure 3C because LAMP-1-RFP is lysosomal membrane protein. Figure S3 complements Figure 3C and supports the possibility that soluble component in His16-Lyso was transported into endogenous lysosomes.

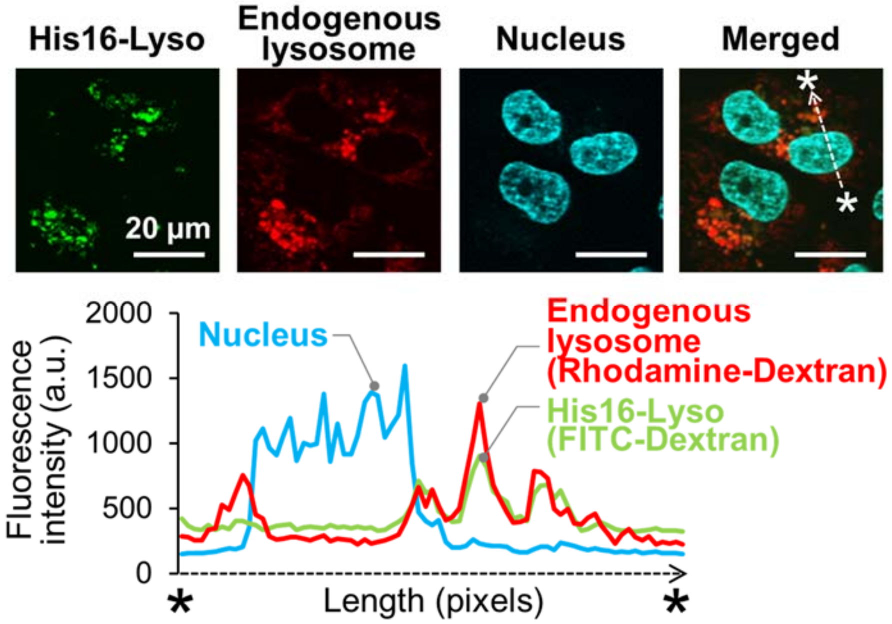


**Figure S3.** Intracellular distribution of His16-Lyso containing FITC-dextran in HT1080 cells. Green, red, and cyan fluorescence indicates His16-Lyso containing FITC-dextran, endogenous lysosomes stained with rhodamine-dextran, and nuclei stained with Hoechst 33258, respectively. The distributions of His16-Lyso, endogenous lysosomes, and nuclei were quantified by measurement of the fluorescence intensities along the line between the two asterisks.

**4. ERT efficacy of a large amount of His16-Lyso in FD patient fibroblasts**

FD patient fibroblasts were treated with a large amount of His16-Lyso (25 μg/mL lysosomes). The ERT efficacy against FD patient fibroblasts was evaluated by measuring cell proliferation recovery. Compared with normal fibroblasts, FD patient fibroblasts showed decreased cell proliferation. On the other hand, a large amount of unmodified lysosome restored half of the cell proliferation. This result suggests unmodified lysosomes may have ERT efficacy against FD patient fibroblasts. This possibility can be explained by the result in Figure 5A that unmodified lysosomes showed small cellular uptake in FD patient fibroblasts though no significant differences in comparison with control. However, a large amount of His16-Lyso treatment completely restored the cell proliferation of FD patient fibroblasts (Figure S4), while Figure 6 showed a small amount of His16-Lyso (5 μg/mL lysosomes) restored a half of cell proliferation. This result also supports that His16-Lyso replenished GLA in FD patient fibroblasts and showed ERT efficacy against FD patient fibroblasts.

Furthermore, to excludes the possibility that His16 or STR-His16 peptides affect cell proliferation of FD patient fibroblasts, we determined the influence of His16 and STR-His16 peptides alone on cell proliferation under the same condition in Figure 6 except for the presence of exogenous lysosomes. Cell proliferation assay showed that His16 or STR-His16 peptides show no effect on cell proliferation of FD patient fibroblasts (Figure S5). This result supports that the influences of His16 and STR-His16 peptides on cell proliferation are insignificant.

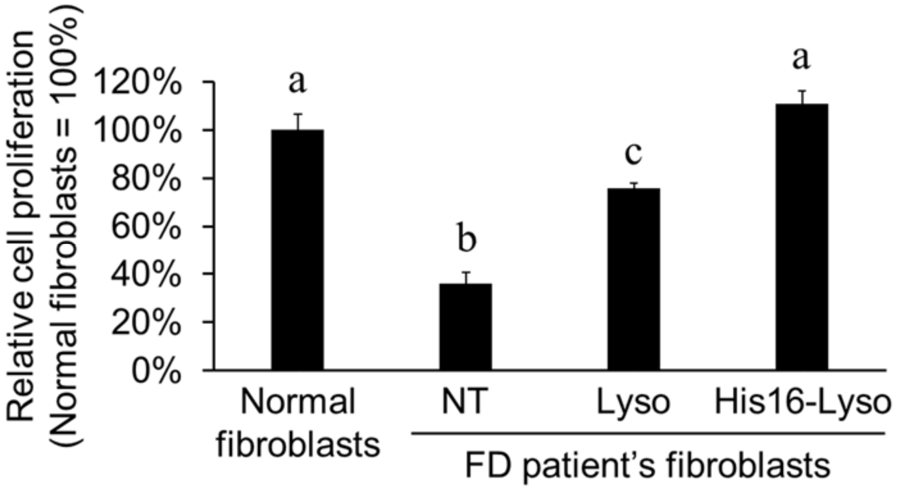


**Figure S4.** ERT efficacy of a large amount of His16-Lyso against FD patient fibroblasts. The FD patient fibroblasts were treated with or without His16-Lyso (25 μg/mL lysosomes) for 72 h at 37°C under 5.0% $CO_2$. NT and Lyso mean nontreated condition and unmodified lysosomes from HT1080 cells, respectively. Cell proliferation was determined by the WST assay. Data represent means ± SD. Different letters indicate significant differences ($p < 0.05$, Tukey's test).

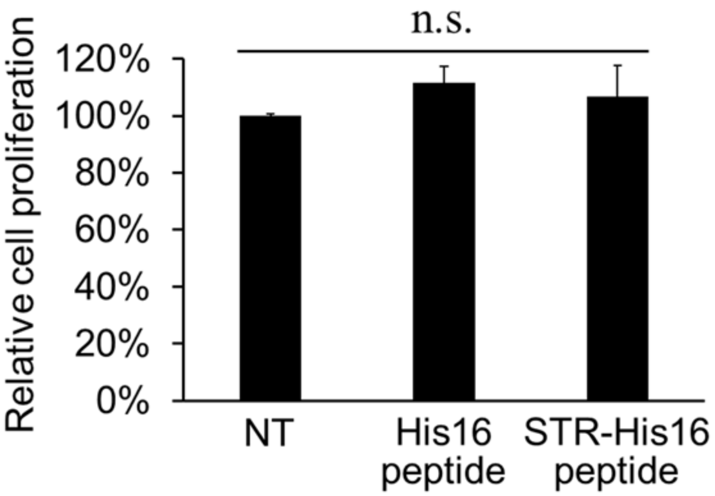


**Figure S5.** Cell proliferation of FD patient fibroblasts treated with His16 or STR-His16 peptides. The FD patient fibroblasts were treated with 10 μM of His16 or STR-His16 peptides for 72 h at 37°C under 5.0% $CO_2$. NT means nontreated condition. Cell proliferation was determined by the WST assay. Data represent means ± SD. n.s. indicates no significant differences in Tukey's test.

© 2019 by the authors. Submitted for possible open access publication under the terms and conditions of the Creative Commons Attribution (CC BY) license (http://creativecommons.org/licenses/by/4.0/).
